# Effective Treatment With Afatinib of Lung Adenocarcinoma With Leptomeningeal Metastasis Harboring the Exon 18 p.G719A Mutation in the EGFR Gene Was Detected in Cerebrospinal Fluid: A Case Report

**DOI:** 10.3389/fonc.2020.01635

**Published:** 2020-09-08

**Authors:** Chunhua Ma, Shuyuan Wang, Ning Mu, Jinduo Li, Mei Liu, Lin Li, Rong Jiang

**Affiliations:** ^1^Tianjin Key Laboratory of Cerebrovascular and Neurodegenerative Disease, Department of Intervention, Tianjin Huanhu Hospital, Tianjin, China; ^2^Tianjin Key Laboratory of Cerebrovascular and Neurodegenerative Disease, Department of Neurosurgery, Tianjin Huanhu Hospital, Tianjin, China

**Keywords:** lung neoplasms, leptomeningeal metastasis, cerebrospinal fluid, non-classical mutations of EGFR, G719A mutation

## Abstract

**Background:** In patients with lung adenocarcinoma and leptomeningeal metastases, it remains unknown whether non-classical mutations in the epidermal growth factor receptor (EGFR) gene can be detected in the cerebrospinal fluid (CSF) and how it may be used to design directed therapy.

**Methods:** On April 18, 2018, the Interventional Department of Tianjin Huanhu Hospital admitted a 34-years-old male patient with lung adenocarcinoma and leptomeningeal metastasis. An emergency lateral ventriculoperitoneal shunt was performed to relieve the clinical symptoms of intracranial hypertension. Next-generation sequencing (NGS) of the CFS specimens revealed a mutation in EGFR exon 18 p.G719A, and afatinib was administered. Follow-up showed significantly relieved headache, with significantly reduced soft leptomeningeal abnormal enhancement as revealed by enhanced magnetic resonance imaging and significantly smaller tumors in the left lung by chest computed tomography. Carcinoembryonic antigens (CEAs) in cerebrospinal fluid and peripheral blood were significantly reduced. The patient responded well to afatinib, with mild adverse complications. The patient died on October 27, 2019 from respiratory failure as a result of lung infection unrelated to cancer progression. The overall survival (OS) using afatinib was 530 days.

**Conclusion:** CSF can be used as a liquid biopsy for NGS gene detection in patients with lung adenocarcinoma and leptomeningeal metastases. Afatinib exhibits a beneficial effect in patients with lung adenocarcinoma and leptomeningeal metastases harboring the EGFR exon 18 p.G719A mutation.

## Introduction

The incidence of leptomeningeal metastasis in patients with non-small cell lung cancer (NSCLC) is about 3.4–3.8% (1), with unfavorable prognosis and median survival in untreated patients of merely 4–6 weeks (2). Lung adenocarcinoma is the pathological type with the highest risk of brain and leptomeningeal metastases. About 43.0–70.5% of lung adenocarcinomas are associated with a sensitive mutation in the epidermal growth factor receptor (EGFR) gene. These patients can receive EGFR tyrosine kinase inhibitors (TKIs) treatment and then exhibit significantly prolonged survival (3), highlighting the importance of determining the mutation status of the EGFR gene. Previous work has shown that the cerebrospinal fluid (CSF) is an effective specimen to complement gene detection in patients with lung adenocarcinoma and leptomeningeal metastasis, from whom pathological tissue samples are not readily accessible for gene detection (4, 5).

The non-classical point mutation G719X is the most common mutation type in the exon 18 of the EGFR gene. Previous work showed that the second generation of EGFR-TKIs are effective in treating lung adenocarcinoma with the EGFR exon 18 p.G719X point mutation (6). In this paper, we report a patient with lung adenocarcinoma and leptomeningeal metastasis after multiple cycles of chemotherapy. The EGFR exon18 p.G719A gene mutation was detected in the CSF (Lizhu Gene, NGS), and a beneficial clinical effect was observed after afatinib treatment. The current findings, therefore, provide a reference for the treatment of lung adenocarcinoma with leptomeningeal metastasis harboring the EGFR exon 18 p.G719A mutation.

## Case Report

This was reviewed and approved by the ethics committee of Tianjin Huanhu Hospital. Written informed consent was obtained from the patient for publication of this case report and any potentially identifying information and images.

A 34-years-old man with an intractable headache for 6 months and blindness for 3 weeks visited our hospital on April 18, 2018. The patient was hospitalized in May 2016 at another hospital for chest tightness, and computed tomography (CT) revealed space-occupying lesions in the left lower lung, indicative of peripheral lung cancer, and this was confirmed as lung adenocarcinoma by subsequent bronchoscopy. Genetic testing of the tumor specimen was performed, and it was found that the EGFR G719A mutation had an abundance of 0.12%. No targeted therapy was given, and six cycles of intravenous docetaxel combined with cisplatin were administered, leading to partial remission. In April 2017, chest CT suggested progression of the left lung tumor, and 10 cycles of intravenous pemetrexed combined with carboplatin were administered, with the last dose in December 2017. During chemotherapy, the patient developed intractable dizziness with nausea and vomiting.

Magnetic resonance imaging (MRI) in June 2017 suggested leptomeningeal metastasis, with tumor cells detected in CSF cytology. Combined with oral temozolomide, whole-brain radiotherapy (WBRT) was performed at a dose of 2 Gy/15 f, for a total of 30 Gy. Peripheral blood mutation detection (microdrip digital PCR assay) was performed in September 2017, but no sensitive mutations of the EGFR gene were detected. In October 2017, the patient suffered from dizziness and severe headache, and the dose of intravenous mannitol was increased gradually to 250 ml q 6 h (drip) with bevacizumab and semustine, but the headache and nausea were not relieved. Three weeks before admission, the patient developed blindness and was admitted to the hospital with a diagnosis of lung adenocarcinoma with leptomeningeal metastasis. The patient was normal since the disease onset in terms of mentality, sleep, poor diet, and feces, with a weight loss of 10 kg. The patient had no previous smoking or alcohol drinking history or family members with tumor history. After admission, the patient showed disorganized consciousness and poor mental status, with the eyes showing no light reflex and a bilateral pupil at about 5 mm. The leptomeningeal stimulation sign was positive. Palpation of the left clavicle area revealed many 1-cm swollen lymph nodes with poor movement and unclear boundaries. There was also a rale sound when the patient breathed. Limb muscle strength was level IV. The patient was diagnosed with stage IV left pulmonary peripheral lung cancer, T3N3M1c (leptomeningeal and lymph node metastasis), and Eastern Collaborative Oncology Group (ECOG) performance status rating of 4. Lung biopsy could not be performed at that time because of the poor condition of the patient.

**Figure 1:**
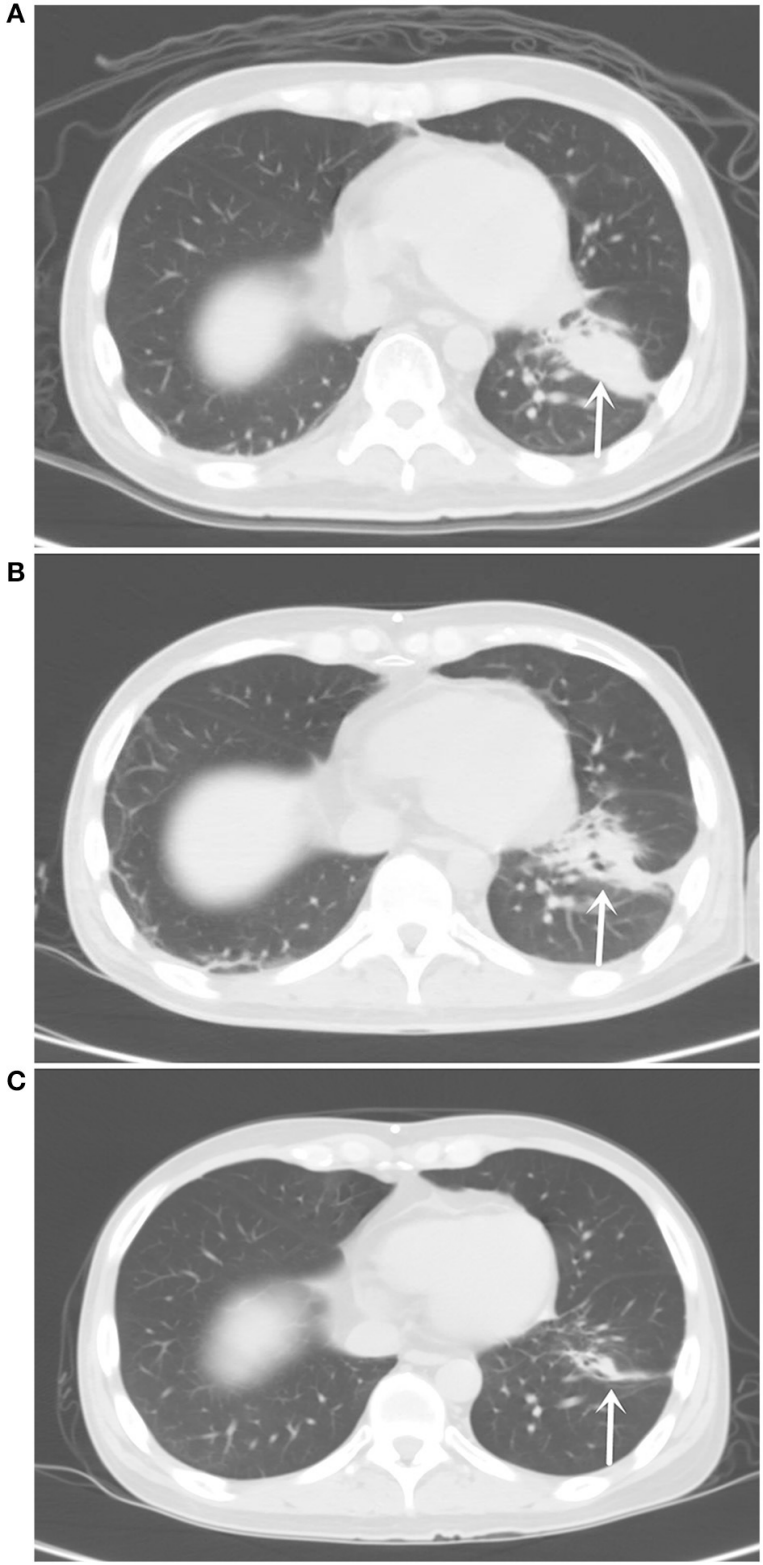
**(A)** Chest computed tomography (CT) pulmonary window on April 19, 2018 showed an irregular solid mass in the lower lobe of the left lung of about 4.0 × 1.9 cm in size. **(B)** Chest CT pulmonary window on July 25, 2018 showed the tumor in the lower lobe of the left lung with an irregular shape and uneven density at about 3.5 × 1.1 cm. **(C)** Chest CT pulmonary window on September 18, 2019 showed an irregular solid mass striated shadow in the lower lobe of the left lung at 2.1 × 0.4 cm in size.

CT examination on April 19, 2018 showed expansion of the supratentorial ventricle system and widening of the sulcus cerebri and cistern. Chest CT on April 19, 2018 revealed an irregular solid mass in the lower lobe of the left lung, about 4.0 × 1.9 cm in size, considered as peripheral lung cancer (Figure 1A). Blood carcinoembryonic antigen (CEA) was 716.8 ng/ml. The CSF examination pressure of lumbar puncture was 272 mmH_2_O (1 mmH_2_O = 9.81 × 10^−3^ kPa, normal reference value of 80–180 mmH_2_O). A right lateral ventriculoperitoneal shunt (VPS) was performed under general anesthesia on April 21, 2018. Lumbar puncture was performed on April 25, 2018. CSF pressure was 240 mmH_2_O. The patient had an intractable headache. Symptoms of limb convulsions were alleviated earlier. After intrathecal chemotherapy (methotrexate 8 mg and dexamethasone 1 ml added to 9 ml of normal saline) on May 8, 2018, the CSF pressure was 109 mmH_2_O.

**Figure 2:**
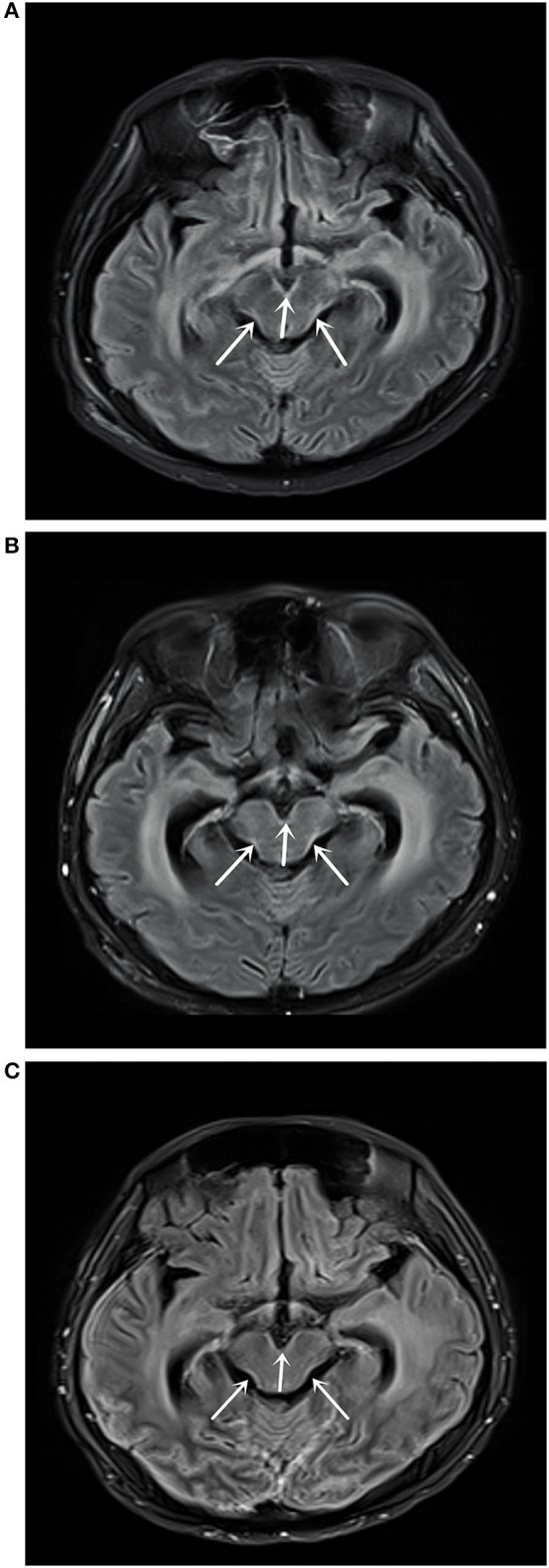
**(A)** Magnetic resonance imaging (MRI) T2 fluid-attenuated inversion recovery (FLAIR) enhanced scan on May 10, 2018 showed abnormally high signal shadows in the medulla oblongata, pons, and ventral and dorsolateral line of the midbrain, suggesting soft leptomeningeal metastasis. **(B)** MRI T2 FLAIR enhanced scan on July 31, 2018 showed medulla oblongata, pons, midbrain ventral, and dorsolateral line abnormally high signal shadows, with no significant changes compared with the MRI results on May 10, 2018. **(C)** MRI T2 FLAIR enhanced scan on September 18, 2019 showed medulla oblongata, pons, midbrain ventral, and dorsal lateral abnormally high signal shadows.

For the liquid biopsy, 10 ml of CSF was slowly drained using a puncture needle, and 10 ml of peripheral blood was drawn. Both specimens were examined for mutations using next-generation sequencing (NGS). CSF CEA levels were 9,470 ng/ml, and, together with grade 3 bone marrow suppression, intrathecal chemotherapy was terminated. On May 10, 2018, an enhanced MRI scan showed abnormal high-signal images of the medulla oblongata, pontine, ventral midbrain, and dorsal lateral lineaments, indicative of leptomeningeal metastasis (Figure 2A). On May 15, 2018, the EGFR exon 18 p.G719A mutation was identified in CSF specimens, with a frequency of 55.6%. No EGFR gene sensitive mutation was detected in the peripheral blood. The cell-free DNA (cfDNA) platform can also detect EGFR p.G873R, FLT3 p.K602R, KIT p.I841T, NRAS p.G12V, and TP53 p.M243V, but they were all negative. The patient received afatinib 30 mg/day. One week later, the headache symptoms were significantly relieved, but the binocular blindness remained. An ophthalmologist was consulted, who considered this as optic nerve injury that was not recoverable. Afatinib 30 mg/day was continued.

On July 25, 2018, CT examination showed that the lower lobe of the left lung was irregular in shape and uneven in density with a size of about 3.50 × 1.1 cm (Figure 1B). Enhanced MRI scan on July 31, 2018 showed a high signal shadow of the medulla oblongata, pontine, ventral midbrain, and dorsal lateral lineation, with no significant changes compared with the previous one (Figure 2B). The CEA levels in peripheral blood and CSF were 126.7 and 2,111.0 ng/ml, respectively. On August 10, 2018, the re-examination of the CSF showed the EGFR exon 18 p.G719A mutation with a frequency of 22.2%. The patient was considered as partial remission (PR) according to Response Evaluation Criteria in Solid Tumors (RECIST) v1.0. No major adverse drug reactions were observed, except mild facial skin rash. The headache symptoms were significantly alleviated, with ECOG at 2. Afatinib was increased to 40 mg/day, and no intolerant drug adverse reactions occurred. After that, the tumor size was stable when examined every 8 weeks. On November 20, 2018, the EGFR exon 18 p.G719A mutation frequency in the CSF was 23.1%. The CEA levels were 92.99 ng/ml in the peripheral blood and 1,590.0 ng/ml in CSF.

**Figure 3:**
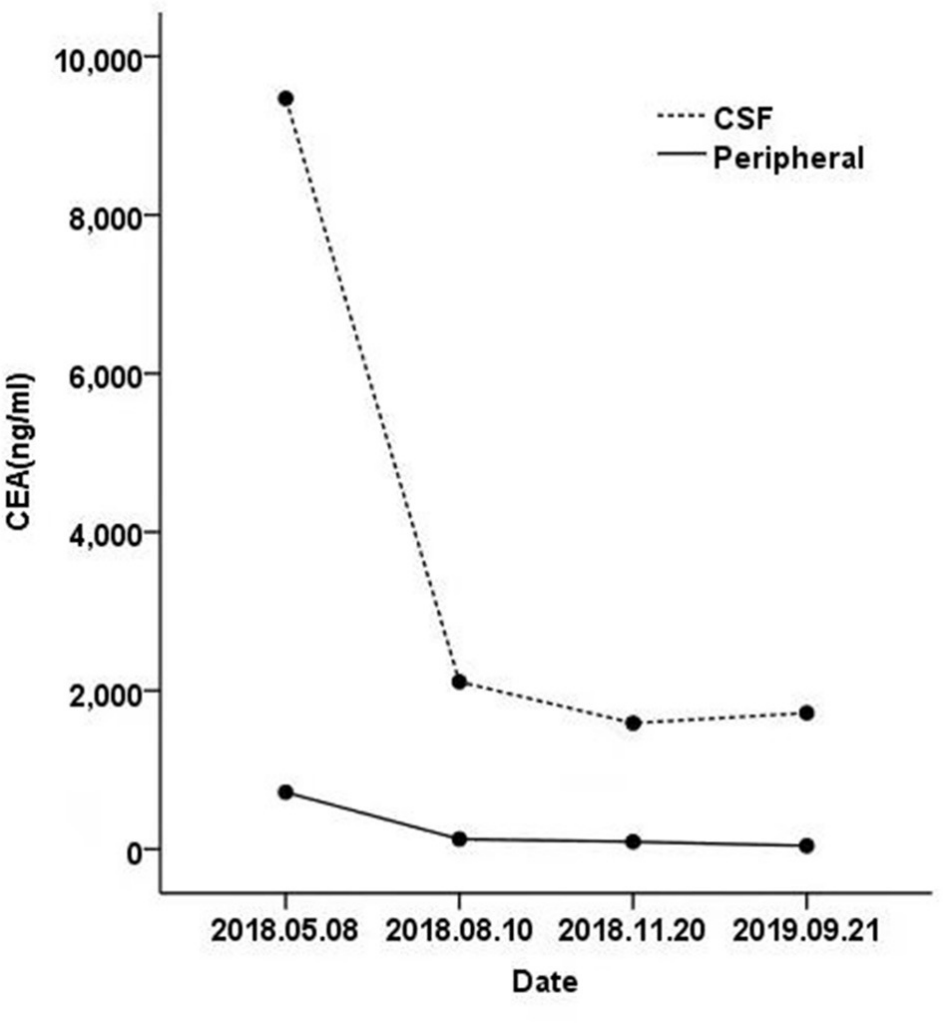
The carcinoembryonic antigen (CEA) levels from the cerebrospinal fluid (CSF) and peripheral blood were significantly decreased.

The last re-examination was on September 18, 2019. Chest CT showed an irregular solid mass in the lower lobe of the left lung, about 2.1 × 0.4 cm in size (Figure 1C). An enhanced MRI scan showed an abnormally high signal shadow on the ventral and dorsal side of the medulla oblongata, pontine, and midbrain. The abnormal signal showed no significant change compared with the previous one (Figure 2C). Genetic examination of the CSF on September 21, 2019 showed the EGFR exon 18 p.G719A mutation with a frequency of 25.5% (Table 1). The CEA levels in the peripheral blood and CSF were 42.95 and 1,717.5 ng/ml, respectively (Figure 3). The patient continued to be treated with afatinib 40 mg/day.

**Table 1:** Genetic mutations detected by liquid biopsy of cerebrospinal fluid (CSF).

**Date**	**Gene**	**Amino acid site**	**Allele frequency**
2018/5/8	EGFR	Exon18 p.G719A	55.6%
2018/8/10	EGFR	Exon18 p.G719A	22.2%
2018/11/20	EGFR	Exon18 p.G719A	23.1%
2019/9/21	EGFR	Exon18 p.G719A	25.5%

On October 27, 2019, the patient died of respiratory failure due to lung infection unrelated to tumor progression. The progression-free survival (PFS) of the patient upon afatinib treatment was 530 days.

## Discussion

Leptomeningeal metastases are serious lesions caused by malignant tumor cells that enter the bloodstream, the lymphatic system, and the CSF and implant themselves into the leptomeninges. Alternatively, adjacent tumors may directly invade the leptomeninges. In both cases, this leads to clinical damage to the brain parenchyma, the brain nerves, and the spinal cord. The clinical manifestations are overall complex and varied, and early diagnosis is difficult using the routine examination. Current treatments for lung cancer with leptomeningeal metastases include WBRT, surgery, drug chemotherapy, and molecular targeted drug therapy (3). The primary objective is to improve or stabilize the central nervous system functions and relieve the symptoms. Nevertheless, the clinical outcomes of those treatment strategies remain unsatisfactory, with a median survival time of only 4–6 months. In this study, given the intractable dizziness symptoms with nausea and vomiting, enhanced MRI revealed abnormal enhancement of the leptomeninges, and CSF cytology identified tumor cells, indicative of lung adenocarcinoma with leptomeningeal metastasis. Pemetrexed combined with temozolomide, WBRT, and other comprehensive treatment led to a stable clinical condition for only 4 months. The clinical symptoms aggravated again, although mannitol dehydration was used to reduce intracranial pressure. Bevacizumab, semustine, and other drugs were also tried, but the beneficial effect was modest. When admitted to our emergency department, the clinical condition of the patient was poor, with intractable headache, binocular blindness, limb convulsion, consciousness loss, and ECOG performance status rating of 4. The expected survival time was <4 weeks. CT scan upon admission showed significant ventricular dilatation, cerebral stroma edema, and significantly increased CSF pressure (272 mmH_2_O) that required an acute right lateral ventriculoperitoneal shunt. Subsequent re-examination showed gradually decreasing CSF pressure. The intractable headache became gradually alleviated. A lateral ventricle–peritoneal shunt can improve encephalopathy resulting from hydrocephalus by effectively draining CSF and lowering CSF pressure in patients with significantly elevated CSF pressure (7, 8).

EGFR-TKIs hold promise for patients with central nervous system metastases from lung adenocarcinoma with a sensitive mutation in the EGFR gene. EGFR-TKIs are fat-soluble, small molecular weight drugs with a capacity to penetrate the blood–brain barrier. In patients with leptomeningeal metastases from non-small cell lung cancer with EGFR gene-sensitive mutations, beneficial results can be obtained using EGFR-TKIs (9, 10). EGFR gene mutations are found mainly in exons 18, 19, 20, and 21, among which the frequency of the deletion in exon 19 and the L858R point mutation in exon 21 is 90%. They are considered as classic sensitive mutation, while the others are considered as non-classical mutation (6, 11). About 3.6% of EGFR mutations are exon 18 mutations, especially for the G719X.719 mutation where the glycine is substituted for an alanine (G719A), cysteine (G719C), or serine (G719S). Prior studies have shown that this mutation increases the distance between residual L718 and G796, making ATP-binding pockets more open and thus easier to bind to TKIs (11, 12). *In vitro* studies showed that exon 18, a driver mutation, was more sensitive to second-generation EGFR-TKIs than to the second and third generations (13). Patients with the G719X mutation treated with second-generation EGFR-TKIs exhibited a median PFS >12 months and an objective response rate of 78% (6). Fan et al. (4) demonstrated consistent information of EGFR gene mutation when using primary tumor vs. CSF specimens from 11 patients with lung adenocarcinoma and leptomeningeal metastasis, suggesting that CSF liquid biopsies are an effective complement for gene mutation detection when pathological specimens are not readily accessible. Li et al. (5) compared the CSF cell-free DNA (cfDNA), CSF precipitate, and plasma samples from 26 patients with lung adenocarcinoma and leptomeningeal metastases using NGS and found that the sensitivity of driving gene mutation was 100% (26/26), 84.6% (22/26), and 73.1% (19/26), respectively. Compared with plasma and primary tumor tissue, CSF cfDNA exhibits unique gene profiles. Leptomeningeal metastasis from non-small cell lung cancer is considered to be the most representative fluid biopsy in which EGFR gene-sensitive mutations can be identified. The 2018 Consensus for Diagnosis and Treatment of Brain (Leptomeningeal) Metastasis of Lung Cancer in China recommends that CSF gene detection be performed in patients with lung adenocarcinoma and leptomeningeal metastases. In the case reported here, the patient was in a highly unfavorable condition when admitted, and intrathecal chemotherapy had to be terminated due to bone marrow suppression. Consequently, mutation detection from CSF and blood was performed. The results indicated the EGFR exon 18 p.G719A mutation with a mutation frequency of 55.6% in CSF, but the results from the blood samples were negative. Bai et al. (14) showed that intravenous chemotherapy reduced the frequency of EGFR gene mutations. The patient underwent two lines of platinum-containing intravenous chemotherapy, oral temozolomide, and semustine chemotherapy, and the lung lesions were stable, but leptomeningeal metastasis showed progression. We, therefore, speculate that the negative gene test results of the blood sample may stem from the patient's multicycle chemotherapy history and the effective control of the lung lesions. Nevertheless, because of the blood–brain barrier and the progression of the leptomeningeal metastasis, the mutation could be detected in CSF specimens. Gene detection using CSF and peripheral blood in patients with lung adenocarcinoma and leptomeningeal metastasis was demonstrated by Huang et al. (15), who also suggested a higher positive detection rate using CSF than peripheral blood.

We found for the first time that the mutation frequency of the EGFR G719A mutation in CSF remained the same (Table 1), although the clinical symptoms improved significantly during afatinib treatment. We believe that in patients with leptomeningeal metastases from lung adenocarcinoma, CSF NGS bears the following characteristics. Foremost, because of the blood–brain barrier that restricts the value of plasma cfDNA or circulating tumor cells in evaluating intracranial lesions, the use of CSF specimens better reflects the gene spectrum of brain tumor lesions. Second, cfDNA from tumor source in plasma could exhibit background interference, whereas this interference is smaller in CSF. The high mutation frequency can be detected by NGS. Third, CSF gene detection may be used to dynamically monitor tumor load in leptomeningeal metastases, thereby revealing the efficacy and prognosis for EGFR-TKIs. Therefore, CSF NGS may have advantages over the unique gene spectrum of the reactive intracranial lesions, and this will help determine whether lung adenocarcinoma patients with leptomeningeal metastases can be treated with EGFR-TKIs or not.

CSF liquid biopsy for the detection of driver mutation in NSCLC leptomeningeal metastases is relatively novel. The clinical significance of uncommon EGFR mutations on the development of leptomeningeal metastases from NSCLC and their response to treatment remains unclear. CSF liquid biopsy has emerged recently as a novel tool for assessing EGFR mutations in intracranial metastases. A recent study showed that in patients with NSCLC and leptomeningeal metastasis, the rate of uncommon EGFR mutation was high and that the brain metastases with uncommon EGFR mutations seem to respond to EGFR-TKI treatment (16). CSF liquid biopsy could reveal the EGFR genetic profile of the BM and help guide treatment using small-molecule TKI. Molecular testing of CSF could be helpful in guiding treatment and tracking treatment response. Indeed, previous studies reported that the EGFR mutations found in the NSCLC primary lesions are not consistent with those found in the intracranial metastases (17–19). Indeed, a primary tumor is composed of multiple different clones (20, 21), and not all of them will have the abilities to spread in circulation, cross the blood–brain barrier, survive in the brain microenvironment, and invade brain tissues (22, 23). Those characteristics require specific sets of factors and mutations, and uncommon mutations might be considered as participating in the process of brain metastases of NSCLC. CSF liquid biopsy could be a new tool for the management of NSCLC, especially in the context where afatinib is able to cross the blood–brain barrier in sufficient amounts to induce an antitumor effect (24, 25).

Guided by the CSF NGS results, the patient received oral afatinib therapy. The intractable headaches were significantly relieved, with no intolerable adverse drug reactions. A chest CT examination showed a significant reduction in lung lesions. An enhanced MRI scan of the head showed the reduced intensity of leptomeningeal lesions and CEA in both CSF and peripheral blood. The patient died of respiratory failure due to lung infection unrelated to tumor progression. The OS was 530 days after starting apatinib, suggesting a beneficial clinical effect for afatinib on leptomeningeal metastasis resulting from lung adenocarcinoma and harboring the EGFR exon 18 p.G719A mutation.

## Ethics Statement

The studies involving human participants were reviewed and approved by Tianjin Huanhu Hospital. The patients/participants provided their written informed consent to participate in this study.

## Author Contributions

CM and SW carried out the studies, participated in collecting the data, and drafted the manuscript. RJ performed the statistical analysis and participated in its design. NM, JL, ML, and LL helped draft the manuscript. All authors read and approved the final manuscript.

## Conflict of Interest

The authors declare that the research was conducted in the absence of any commercial or financial relationships that could be construed as a potential conflict of interest.

## References

[1] Jiang BY Li YS Guo WB Zhang XC Chen ZH Su J. Detection of driver and resistance mutations in leptomeningeal metastases of NSCLC by next-generation sequencing of cerebrospinal fluid circulating tumor cells. Clin Cancer Res. (2017) 23:5480–8. 10.1158/1078-0432.Ccr-17-0047 28606923

[2] Ma C Jiang R Li J Wang B Sun L Lv Y. Research progress of lung cancer with leptomeningeal metastasis. Zhongguo Fei Ai Za Zhi. (2014) 17:695–700. 10.3779/j.issn.1009-3419.2014.09.10 25248713 PMC6000511

[3] Xu Y Li L Wang M. Diagnosis and treatment of leptomeningeal metastasis in non-small cell lung cancer. Zhongguo Fei Ai Za Zhi. (2015) 18:626–32. 10.3779/j.issn.1009-3419.2015.10.05 26483335 PMC6000085

[4] Fan Y Zhu X Xu Y Lu X Xu Y Wang M. Cell-cycle and DNA-damage response pathway is involved in leptomeningeal metastasis of non-small cell lung cancer. Clin Cancer Res. (2018) 24:209–16. 10.1158/1078-0432.Ccr-17-1582 29030356

[5] Li YS Jiang BY Yang JJ Zhang XC Zhang Z Ye JY. Unique genetic profiles from cerebrospinal fluid cell-free DNA in leptomeningeal metastases of EGFR-mutant non-small-cell lung cancer: a new medium of liquid biopsy. Ann Oncol. (2018) 29:945–52. 10.1093/annonc/mdy009 29346604

[6] Yang JC Sequist LV Geater SL Tsai CM Mok TS Schuler M. Clinical activity of afatinib in patients with advanced non-small-cell lung cancer harbouring uncommon EGFR mutations: a combined *post-hoc* analysis of LUX-Lung 2, LUX-Lung 3, and LUX-Lung 6. Lancet Oncol. (2015) 16:830–8. 10.1016/s1470-2045(15)00026-1 26051236

[7] Lee SH Kong DS Seol HJ Nam DH Lee JI. Ventriculoperitoneal shunt for hydrocephalus caused by central nervous system metastasis. J Neurooncol. (2011) 104:545–51. 10.1007/s11060-010-0512-2 21274592

[8] Lee SJ Lee JI Nam DH Ahn YC Han JH Sun JM. Leptomeningeal carcinomatosis in non-small-cell lung cancer patients: impact on survival and correlated prognostic factors. J Thorac Oncol. (2013) 8:185–91. 10.1097/JTO.0b013e3182773f21 23328548

[9] Yi HG Kim HJ Kim YJ Han SW Oh DY Lee SH Epidermal growth factor receptor (EGFR) tyrosine kinase inhibitors (TKIs) are effective for leptomeningeal metastasis from non-small cell lung cancer patients with sensitive EGFR mutation or other predictive factors of good response for EGFR TKI. Lung Cancer. (2009) 65:80–4. 10.1016/j.lungcan.2008.10.016 19059670

[10] Lee E Keam B Kim DW Kim TM Lee SH Chung DH. Erlotinib versus gefitinib for control of leptomeningeal carcinomatosis in non-small-cell lung cancer. J Thorac Oncol. (2013) 8:1069–74. 10.1097/JTO.0b013e318294c8e8 23804027

[11] Wang Y Li M Hu CP Rare mutation and targeted therapy of EGFR gene in non-small cell lung cancer. Natl Med J China. (2019) 99:154–7. 10.3760/cma.j.issn.0376-2491.2019.02.016

[12] Tamiya M Shiroyama T Nishihara T Nishida T Hayama M Tanaka A Afatinib successfully treated leptomeningeal metastasis during erlotinib treatment in a patient with EGFR-mutant (Exon18:G719S) lung adenocarcinoma as a second-line chemotherapy. Asia Pac J Clin Oncol. (2017) 13:e531–3. 10.1111/ajco.12643 28004883

[13] Banno E Togashi Y Nakamura Y Chiba M Kobayashi Y Hayashi H. Sensitivities to various epidermal growth factor receptor-tyrosine kinase inhibitors of uncommon epidermal growth factor receptor mutations L861Q and S768I: what is the optimal epidermal growth factor receptor-tyrosine kinase inhibitor? Cancer Sci. (2016) 107:1134–40. 10.1111/cas.12980 27240419 PMC4982590

[14] Bai H Wang Z Chen K Zhao J Lee JJ Wang S. Influence of chemotherapy on EGFR mutation status among patients with non-small-cell lung cancer. J Clin Oncol. (2012) 30:3077–83. 10.1200/jco.2011.39.3744 22826274 PMC5321076

[15] Huang R Xu X Li D Chen K Zhan Q Ge M. Digital PCR-based detection of EGFR mutations in paired plasma and CSF samples of lung adenocarcinoma patients with central nervous system metastases. Target Oncol. (2019) 14:343–50. 10.1007/s11523-019-00645-5 31161597

[16] Ma C Zhang J Tang D Ye X Li J Mu N. Tyrosine kinase inhibitors could be effective against non-small cell lung cancer brain metastases harboring uncommon EGFR mutations. Front Oncol. (2020) 10:224. 10.3389/fonc.2020.00224 32195178 PMC7066117

[17] Burel-Vandenbos F Ambrosetti D Coutts M Pedeutour F. EGFR mutation status in brain metastases of non-small cell lung carcinoma. J Neurooncol. (2013) 111:1–10. 10.1007/s11060-012-0990-5 23086434

[18] Hata A Katakami N Yoshioka H Takeshita J Tanaka K Nanjo S. Rebiopsy of non-small cell lung cancer patients with acquired resistance to epidermal growth factor receptor-tyrosine kinase inhibitor: comparison between T790M mutation-positive and mutation-negative populations. Cancer. (2013) 119:4325–32. 10.1002/cncr.28364 24105277

[19] Kelly WJ Shah NJ Subramaniam DS. Management of brain metastases in epidermal growth factor receptor mutant non-small-cell lung cancer. Front Oncol. (2018) 8:208. 10.3389/fonc.2018.00208 30018881 PMC6037690

[20] McGranahan N Swanton C. Clonal heterogeneity and tumor evolution: past, present, and the future. Cell. (2017) 168:613–28. 10.1016/j.cell.2017.01.018 28187284

[21] Jia Q Wu W Wang Y Alexander PB Sun C Gong Z. Local mutational diversity drives intratumoral immune heterogeneity in non-small cell lung cancer. Nat Commun. (2018) 9:5361. 10.1038/s41467-018-07767-w 30560866 PMC6299138

[22] Winkler F. Hostile takeover: how tumours hijack pre-existing vascular environments to thrive. J Pathol. (2017) 242:267–72. 10.1002/path.4904 28390068

[23] Preusser M Winkler F Valiente M Manegold C Moyal E Widhalm G. Recent advances in the biology and treatment of brain metastases of non-small cell lung cancer: summary of a multidisciplinary roundtable discussion. ESMO Open. (2018) 3:e000262. 10.1136/esmoopen-2017-000262 29387475 PMC5786916

[24] Hoffknecht P Tufman A Wehler T Pelzer T Wiewrodt R Schutz M Efficacy of the irreversible ErbB family blocker afatinib in epidermal growth factor receptor (EGFR) tyrosine kinase inhibitor (TKI)-pretreated non-small-cell lung cancer patients with brain metastases or leptomeningeal disease. J Thorac Oncol. (2015) 10:156–63. 10.1097/JTO.0000000000000380 25247337 PMC4276567

[25] Schuler M Wu YL Hirsh V O'Byrne K Yamamoto N Mok T. First-line afatinib versus chemotherapy in patients with non-small cell lung cancer and common epidermal growth factor receptor gene mutations and brain metastases. J Thorac Oncol. (2016) 11:380–90. 10.1016/j.jtho.2015.11.014 26823294

